# Changes in otolaryngology application requirements and match outcomes: Are we doing any better?

**DOI:** 10.1002/wjo2.79

**Published:** 2022-10-02

**Authors:** Emma De Ravin, Ariel S. Frost, Neal R. Godse, Amber D. Shaffer, Noel Jabbour, Barry M. Schaitkin, Jason Newman, Leila J. Mady

**Affiliations:** ^1^ Department of Otorhinolaryngology—Head and Neck Surgery University of Pennsylvania Health System Philadelphia Pennsylvania USA; ^2^ Head and Neck Institute Cleveland Clinic Cleveland Ohio USA; ^3^ Division of Pediatric Otolaryngology Children's Hospital of Pittsburgh of UPMC Pittsburgh Pennsylvania USA; ^4^ Department of Otolaryngology University of Pittsburgh Medical Center Pittsburgh Pennsylvania USA; ^5^ Department of Otolaryngology – Head and Neck Surgery Medical University of South Carolina Charleston South Carolina USA; ^6^ MUSC Hollings Cancer Center Charleston South Carolina USA; ^7^ Department of Otolaryngology – Head and Neck Surgery Thomas Jefferson University Philadelphia Pennsylvania USA; ^8^ Cancer Risk and Control Program of Excellence Sidney Kimmel Cancer Center Philadelphia Pennsylvania USA

**Keywords:** academic success, internship and residency, medical students, otolaryngology, surveys and questionnaires

## Abstract

**Objectives:**

Otolaryngology‐specific requirements were piloted to minimize applicant and program burdens. We investigated the impact of introducing and then removing these requirements on Match outcomes.

**Methods:**

2014–2021 National Resident Matching Program® data were examined. The primary outcome was the impact of Otolaryngology Resident Talent Assessment (ORTA; prematch 2017, postmatch 2019) and Program‐Specific Paragraph (PSP; implemented 2016, optional 2018) on applicant numbers and match rates. Secondary survey analysis assessed candidate perceptions of PSP/ORTA.

**Results:**

Applicant numbers declined significantly during PSP/ORTA (18.9%; *p* = 0.001). With the optional PSP and postmatch ORTA, applicant numbers increased significantly (39.0%; *p* = 0.002). Examined individually, mandatory PSP was associated with a significant decline in applicants (*p* = 0.007), whereas postmatch ORTA was associated with significant increases in applicants (*p* = 0.010). ORTA and PSP negatively impacted the decision to apply to otolaryngology in 59.8% and 51.3% of applicants, respectively. Conversely, match rate success improved significantly from 74.8% to 91.2% during PSP/ORTA (*p* = 0.014), followed by a significant decline to 73.1% after PSP was made optional and ORTA moved to postmatch (*p* = 0.002).

**Conclusions:**

ORTA and PSP correlated with decreased applicant numbers and increased match rate success. As programs seek ways to remove barriers to applying to otolaryngology, the potential consequences of an increasing pool of unmatched candidates must also be considered.

## Key points

- The Program‐Specific Paragraph (PSP) and Otolaryngology Resident Talent Assessment (ORTA) were added with good intent—so candidates could convey serious intentions to specific programs (via the PSP) and for programs to identify excellent, “best‐fit” future residents (via the ORTA).
- While PSP and ORTA have been suggested to contribute to declining applicant numbers, the implications of these interventions have not been thoroughly investigated. We evaluated the impact of introducing and then removing these requirements on Match outcomes from 2014 to 2021.
- We found that applicant numbers declined significantly (18.9%) and match rate success increased significantly (16.4%) during PSP/ORTA. When PSP was made optional and ORTA was moved to postmatch, applicant numbers and match rates returned to baseline.
- ORTA and PSP negatively impacted the decision to apply to otolaryngology in 59.8% and 51.3% of applicants, respectively.
- Any new Match interventions must involve a combination of application/selection process reform and pregraduate curriculum development. Additional application requirements must be instituted strategically and with careful consideration for the implications on applicant numbers and match rate success.

## INTRODUCTION

Since its transition from an “early match” coordinated by the San Francisco match to the Main Residency Match® (“the Match”) in 2006, otolaryngology has remained one of the most competitive specialties in medicine.^1^, ^2^ The otolaryngology residency application and selection process is plagued by hyperinflation, wherein applications far outnumber available positions. Among all specialties, otolaryngology has the second highest ratio of graduating medical students ranking it first in the Match compared to available positions in that specialty (ratio: 1.18), second only to plastic surgery (ratio: 1.27).^3^ Under such supply‐demand discord, a proportion of graduating medical students risk an unsuccessful match, perpetuating the reputation that matching into otolaryngology is “impossible” or “near‐impossible.”^4^

Previous studies evaluating the otolaryngology Match have attributed its competitiveness to a complex interplay between applicant factors (e.g., number of programs applicants applied to) and program factors (e.g., United States Medical Licensing ExamⓇ [USMLE] score and Alpha Omega Alpha [AOA] status screening, research requirements).^4^, ^5^, ^6^ In the context of these factors, between 2007 and 2016 otolaryngology applicants' mean USMLE Step 1 scores increased by 10 points (average score 248 in 2016); percent AOA membership increased by more than 5%; and the average number of abstracts, presentations, and publications per applicant more than doubled.^5^, ^7^ Fueled by shotgun approaches to applying, there has been a 250% increase in the mean number of applications‐per‐candidate over the last two decades.^7^, ^8^

In the 2015 Match cycle, the Otolaryngology Program Director Organization (OPDO) required applicants to write a separate paragraph for each program (the Program‐Specific Paragraph or PSP) as a medium for candidates to express their specific interest in a program and reduce the number of applications submitted per applicant, thereby improving match rate success.^9^, ^10^ In the subsequent application year, the Otolaryngology Resident Talent Assessment (ORTA) was implemented as a concurrent prerequisite. The ORTA is a structured, telephone‐based interview developed to assess noncognitive attributes of applicants that are not systematically evaluated through traditional requirements, such as USMLE board exam scores, AOA membership, and letters of recommendation. The ORTA was intended to yield psychometric‐based predictions regarding which applicants would excel as otolaryngologists.^11^

**Figure 1:**
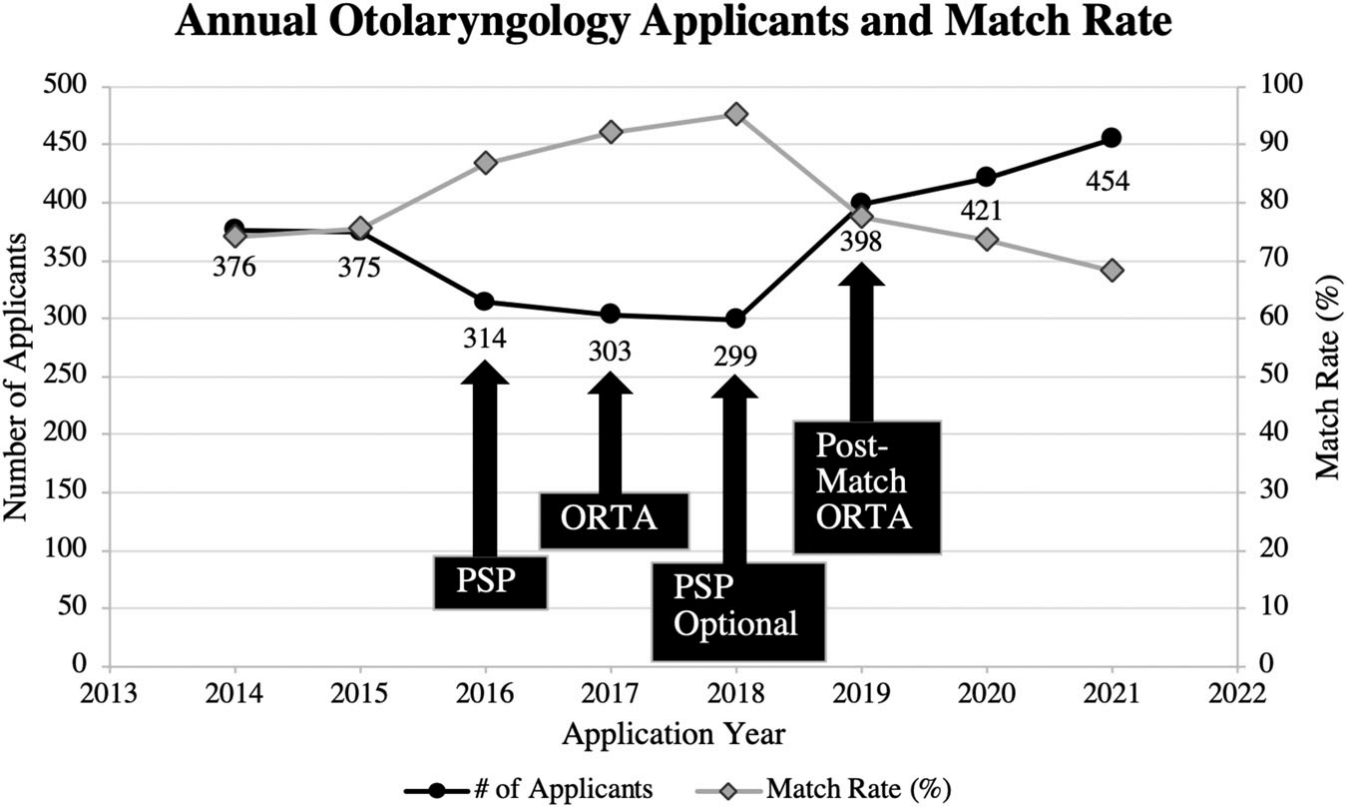
Medical graduate applications to U.S. otolaryngology programs as of 15 February of each year demonstrated a steady decline from 2014 to 2018, and a significant recovery followed by continued growth in 2019–2021. Match rate success followed an opposite trend, with steady growth from 2014 to 2018, and a marked drop followed by a consistent decline from 2019 to 2021. New application requirements including the Program‐Specific Paragraph (PSP) and Otolaryngology Resident Talent Assessment (ORTA) phone interview were implemented in 2016 and 2017, respectively. The PSP became optional in 2018 and starting in 2019, the ORTA was conducted post‐Match.

Since the PSP and ORTA were enacted in 2015 and 2016, respectively, their implementation and characteristics have evolved: the PSP became optional in 2018, and starting in 2019 the ORTA was conducted postmatch. While the PSP and ORTA have been suggested to contribute to declining applicant numbers (Figure 1), the implications of these interventions have not been thoroughly investigated. In this study, we sought to evaluate the impact of introducing and then removing these prematch requirements on Match outcomes between the years 2014–2021. We hypothesized that the introduction of the prematch PSP and ORTA led to a decline in applicant numbers and that medical students perceived the PSP and ORTA as barriers to otolaryngology, contributing to the downward trend observed in applicant numbers.

## MATERIALS AND METHODS

Annual National Resident Matching Program® reports from 2014 to 2021 were examined.^2^ Data collected on application statistics included the number of otolaryngology applicants, number of otolaryngology matches, and match rate for each application year. Continuous variable significance testing was performed in Excel using Student's *t*‐tests between application years.

A secondary survey was designed to assess otolaryngology resident perceptions on the impact of PSP, ORTA phone interview, reputation that it is difficult to match into otolaryngology, number of medical school classmates applying to otolaryngology, and program director (PD) advice on the decision to apply to otolaryngology. In addition, otolaryngology residents were asked about their perceptions of these factors on medical school classmates that considered otolaryngology but applied to another specialty instead (“non‐otolaryngology”). Following Institutional Review Board approval, the survey was circulated to all otolaryngology PDs for distribution to current postgraduate year 1 and 2 (PGY‐1/PGY‐2) otolaryngology residents beginning residency in 2017–2018 or 2018–2019. Survey responses were collected via SurveyMonkey from July to September 2018.

Descriptive statistics were performed in Excel. Fisher's exact test (Stata/SE 13.1; StataCorp) was used to compare responses to questions about the impact of factors (PSP, ORTA phone interview, difficult reputation, and PD advice) on resident decisions to apply to otolaryngology with responses estimating the influence of each of these factors on classmates' decisions to not apply to otolaryngology.

## RESULTS

### Impact on match rates

The number of applicants to otolaryngology declined significantly during the PSP/ORTA period (18.9%; *p* = 0.001), decreasing from a mean of 376 applicants in 2014–2015 (pre‐PSP/ORTA) to 305 in 2016–2018 (during PSP/ORTA); Table 1. When examined individually, only the PSP led to a significant decrease in the number of applicants (17.8%, *p* = 0.007). When the PSP became optional, it did not result in a significant increase in applicants (27.2%; *p* = 0.167), whereas moving the ORTA to postmatch did (40.8%; *p* = 0.010).

**Table 1:** The impact of the PSP and ORTA on the number of applicants to otolaryngology

Item	PSP and ORTA		PSP alone		ORTA alone	
	*n*	*p* Value	*n*	*p* Value	*n*	*p* Value
Prerequirement	376	–	376	–	355	–
During requirement	305	0.001	309	0.007	301	0.134
Optional or postmatch requirement	424	0.002	393	0.167	424	0.010

*Note*: *n* represents mean number of applicants within each of the periods (prerequirement, during requirement, and optional or postmatch requirement).

Abbreviations: ORTA, Otolaryngology Residency Talent Assessment; PSP, Program‐Specific Paragraph; –, no data.

Match rate success followed an opposite trend to applicant numbers. There were significant improvements from 74.8% to 91.2% during PSP/ORTA (*p* = 0.014), followed by significant decline to 73.1% after optional PSP and postmatch ORTA (*p* = 0.002); Table 2. Like applicant numbers, when examined individually, only the PSP led to a significant increase in match rates (PSP 14.6%, *p* = 0.035; ORTA 6.9%, *p* = 0.066). When the ORTA was switched to postmatch and PSP became optional, match rate success decreased by 20% (*p* = 0.011) and 10.6% (*p* = 0.289), respectively.

**Table 2:** The impact of the PSP and ORTA on match rate success

Item	PSP and ORTA		PSP alone		ORTA alone	
	%	*p* Value	%	*p* Value	%	*p* Value
Prerequirement	74.8	–	74.8	–	78.8	–
During requirement	91.2	0.014	89.4	0.035	93.5	0.066
Optional or postmatch requirement	73.1	0.002	78.6	0.289	73.1	0.011

Abbreviations: ORTA, Otolaryngology Residency Talent Assessment; PSP, Program‐Specific Paragraph; –, no data.

### Applicant perceptions

**Figure 2:**
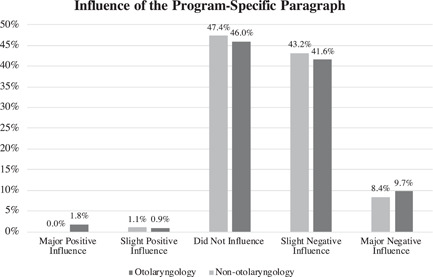
The Program‐Specific Paragraph (PSP) was regarded as a negative influence in 51.3% (*n* = 58/113) of otolaryngology residents (slight negative: 41.6%, *n* = 47/113; major negative: 9.7%, *n* = 11/113). The PSP was estimated to similarly impact nonotolaryngology medical students who applied to a different specialty (slight negative: 43.2%, *n* = 41/95; major negative: 8.7%, *n* = 8/92).

**Figure 3:**
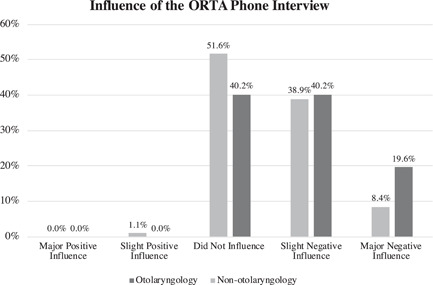
The Otolaryngology Resident Talent Assessment (ORTA) phone interview was regarded as a negative influence in 59.8% (*n* = 64/107) of otolaryngology residents (slight negative: 40.2%, *n* = 43/107; major negative: 19.6%, *n* = 21/107). The ORTA interview was estimated as having a more negative influence on otolaryngology residents than on nonotolaryngology medical students who applied to a different specialty (overall negative: 47.4%, *n* = 45/95; slight negative: 38.9%, *n* = 37/95; major negative: 8.4%, *n* = 8/95; *p* = 0.05).

A total of 118 of 610 (19.3%) otolaryngology residents participated in the survey. Among residents who pursued otolaryngology, 51.3% (*n* = 58/113) regarded the PSP as a negative influence on the decision to apply to otolaryngology, of which 9.7% (*n* = 11) qualified the PSP as a major negative influence (Figure 2). The ORTA phone interview was regarded as a negative influence in 59.8% (*n* = 64/107) of otolaryngology residents, of which 19.6% (*n* = 21) qualified it as a major negative influence (Figure 3). For the classmates who considered otolaryngology but applied to a different specialty, otolaryngology residents estimated that the PSP and ORTA were negative influences in 51.6% (*n* = 49/95) and 47.4% (*n* = 45/95), respectively, of which 8.4% (*n* = 8/95) qualified both the PSP and the ORTA as major negative influences (Figures 2 and 3). Comparing the impact of the PSP and ORTA on otolaryngology residents and the estimated impact on the medical school classmates that considered otolaryngology but did not apply, the PSP impact was similar whereas the ORTA interview was estimated to have a more negative influence on otolaryngology residents than on those who ultimately applied to a different specialty (*p* = 0.050).

**Figure 4:**
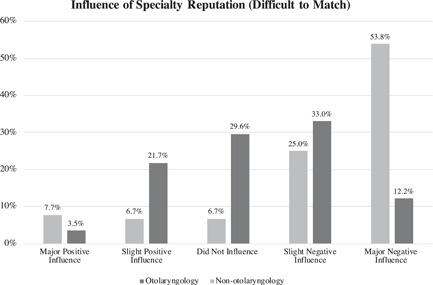
Specialty reputation as difficult to match into was regarded as a negative influence in 45.2% (*n* = 52/115) of otolaryngology residents (slight negative: 33.0%, *n* = 38/115; major negative: 12.2%, *n* = 14/115). Reputation was estimated to have a more negative influence on nonotolaryngology medical students who applied to a different specialty compared to otolaryngology residents (overall negative: 78.8%, *n* = 82/104; slight negative: 25.0%, *n* = 26/104; major negative: 53.8%, *n* = 56/104; *p* < 0.001).

The reputation that it is difficult to match in otolaryngology was viewed as a negative influence among 45.2% (*n* = 52/115) of otolaryngology residents, of which 12.2% (*n* = 14) qualified it as a major negative influence (Figure 4). Reputation was estimated to be a negative influence in 78.9% (*n* = 82/104) of medical students who ultimately applied to a different specialty. Reputation had a stronger negative influence on medical school classmates that considered otolaryngology but did not apply compared to otolaryngology residents (*p* < 0.001).

**Figure 5:**
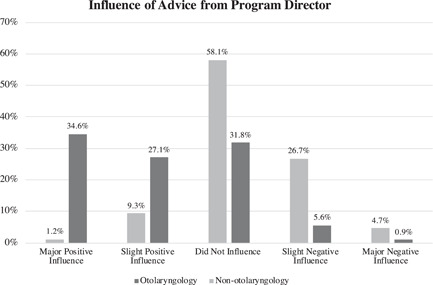
Advice from program directors was viewed as a negative influence in 6.5% (*n* = 7/107) of otolaryngology residents (slight negative: 5.6%, *n* = 6/107; major negative: 0.9%, *n* = 1/107). This advice was estimated to have a more negative influence on nonotolaryngology medical students who applied to a different specialty (overall negative: 31.4%, *n* = 27/86; slight negative: 26.7%, *n* = 23/86; major negative: 4.7%, *n* = 4/86; *p* < 0.001).

Advice from PDs was viewed as a negative influence in 6.5% (*n* = 7/107) of residents who matched into otolaryngology, and 31.4% (*n* = 27/86) of those who applied to a different specialty (Figure 5). Advice from PDs was estimated to have a more negative influence on applicants who applied to other specialties than for the matched otolaryngology residents (*p* < 0.001). Finally, when otolaryngology residents were asked about the influence of the number of classmates applying to otolaryngology from one institution, 69.5% (*n* = 82/118) of residents reported this did not influence their decision to pursue an otolaryngology residency at all, and 18.6% (*n* = 22) regarded this as only a slight negative influence.

## DISCUSSION

Between 2014 and 2018, there was a steady decline in the number of medical students applying to otolaryngology, with a 20% decline in applicants from 376 in 2014 to 299 in 2018.^2^ The underpinnings of this phenomenon are likely multifactorial, with contributions from both applicant and program‐specific factors. Previous studies have focused on specialty competitiveness, with filters pertaining to USMLE board score, AOA membership, and research experience as key factors in the decline in applicants.^4^, ^5^, ^12^ However, as these application qualifications are longstanding, we hypothesized that the prematch PSP and ORTA contributed to the downward trend observed in applicant numbers.

Both the PSP and ORTA were added with good intent—for candidates to convey serious intentions to specific programs and for programs to identify excellent, “best‐fit,” future residents. However, contrary to their intended purpose, this study suggests that both the PSP and ORTA were perceived as barriers to application and led to declines in applicant numbers (and consequent increases in match rate success). When examined individually, however, only the PSP (and not the ORTA) led to a statistically significant decrease in applicant numbers. This effect has been seen previously: when the PSP was first trialed at Duke in the 2014 Match, the program received 25% less applications than in years prior.^9^ Further, the PSP may have had a more negative influence than the ORTA due to qualitative differences. The PSP entails additional research and preparation for each application, a demanding task that compounds with each additional program a candidate applies to, whereas the ORTA is a one‐time 2–3 h time commitment that does not require preparation.

One consequence of declining applicant numbers is the risk of losing high‐quality applicants. Increasing competitiveness (assessed via average USMLE Step 1 score, percent AOA membership, and the number of research experiences) may discourage candidates with unique circumstances, and/or potentially excellent clinicians with below‐average Step 1 scores, who would otherwise make a valuable contribution to the field.^4^, ^5^, ^9^ Further, there is evidence to support that academic achievements like high USMLE Step 1 scores and noteworthy research experience may not predict successful residency performance.^5^, ^13^, ^14^ Although otolaryngology applicants are high‐achieving in each of these domains, more than 90% of programs report having to remediate residents due to unprofessional behavior, insufficient medical knowledge, or poor clinical judgment.^15^ Alternatively, qualities that otolaryngologists do highly value, such as integrity, empathy, and surgical dexterity, are not captured by these academic metrics.^3^

Perceived competitiveness motivates candidates to submit large numbers of applications as a mechanism to increase the likelihood of a successful match.^16^ As a result, over the past two decades, the mean number of applications‐per‐candidate for otolaryngology has increased by nearly 250%.^6^ Among 150 otolaryngology residents surveyed, 90.6% acknowledged applying to programs in which they had no specific interest to improve their chances of matching.^6^ Programs inundated by these application numbers are left grappling to understand candidates' genuine interest in specific programs.

In response to candidates' shotgun approaches to the Match, limitations on the number of applications‐per‐candidate to between 10 and 20 programs have been recommended.^16^ Such restrictions are suggested to enable candidates to focus only on desired programs, decrease interview‐associated travel expenses, and minimize discrepancies in application numbers secondary to financial burden or disadvantage. For residency programs, such constraints would enable reviewers to evaluate applications in greater detail and potentially eliminate selection criteria (such as USMLE score, AOA status, and/or publication numbers) aimed at trimming inflated candidate cohort numbers. With more time to review a smaller pool of applicants, PDs could broaden the evaluation of quantitative criteria (i.e., board and clerkship scores, AOA status) to also include more “humanistic criteria” (i.e., personal accomplishments, letters of recommendation, and personal statements). Several studies have echoed sentiments to implement application limits, albeit discordance remains concerning the specific number that should be permissible; there is currently no method available to limit application numbers.^8^, ^17^

Beyond instituting a limitation on applications‐per‐candidate, numerous proposals have been made to improve the otolaryngology residency application and selection process.^9^ A preference signaling system piloted in 2018 was successfully implemented in the 2021 otolaryngology Match, and will be continued in otolaryngology and appended to dermatology, general surgery, and internal medicine in the 2022 Match cycle.^18^, ^19^ Named “the Star System,” this approach provides each applicant a predetermined number of “stars” or “signals” to send to programs of particular interest.^9^, ^18^ This enables applicants to easily and transparently indicate interest in a select few programs and addresses the current system that leaves programs grappling to understand candidates' genuine interests. Another signaling approach known as the Consortia Match utilizes a hybrid early‐ and conventional‐match system in which residency programs are grouped into “baskets” based upon qualities, including program caliber, reputation, and geography, and applicants are limited to one program “basket” in the early match.^1^, ^9^ By limiting the number of programs a candidate can apply to in the early consortium, this match structure would help reduce strategies such as interview hoarding and improve the match between program and applicant.^1^

The COVID‐19 pandemic further amplified existing concerns about the otolaryngology match process and has led to the implementation of several major structural changes to the otolaryngology match. Beyond preference signaling, these changes included a transition to virtual interviewing, a common interview offer day, and away rotation restrictions. The most impactful, universally implemented, change was the transition to virtual interviews. Without the expenses of traditional in‐person interviews, virtual interviewing reduces the financial cost of interviews (for both programs and applicants).^20^, ^21^, ^22^, ^23^ In this way, virtual interviews may decrease disparities related to financial and geographic circumstances.^24^, ^25^ On the other hand, without the time spent traveling to traditional in‐person interviews, virtual interviewing increases the flexibility of interview scheduling for applicants, perhaps allowing the attendance of additional interviews, which would have been previously constrained by the logistics of travel (“interview hoarding”).^20^, ^24^ In addition, virtual interviewing limits an applicant's ability to evaluate the interpersonal dynamics of a program and assess the “feel” of a geographic area.^20^, ^24^ The virtual model may also introduce additional sources of bias based on an applicant's comfort with technology, internet connection, or perceived environment.^20^

Unlike virtual interviews, which were first implemented in the 2020 Match cycle and have remained through the 2022 Match cycle, recommendations regarding away rotations have been in constant flux. In the 2020 Match cycle, away rotations were banned altogether, while in 2021, students were limited to one away rotation per specialty,^26^ and in 2022, limitations on away rotations were discontinued altogether.^27^ The majority of otolaryngology PDs view away rotations as “extremely important” or “very important” in their evaluation of candidates,^28^ thus away rotation restrictions, though necessary in the pandemic, limited the ability for applicants to familiarize themselves and form a personal relationship with programs other than their home institution. During this period, PDs were forced to shift the ways in which they evaluated candidates (with increased reliance on letters of recommendation, research involvement, and clerkship grades), and students were significantly more likely to match at programs in the state in which they attended medical school.^29^

The third change, a coordinated interview invite release date, was suggested by the OPDO in the 2021 Match cycle, to decrease applicant stress and anxiety surrounding interview scheduling and to improve the efficiency of the interview scheduling process for programs.^26^ Programs were also advised by the OPDO to limit their number of interview invitations to the number of interview slots available.^26^ However, these changes were merely suggestions, not mandates, and thus were not universally implemented.^30^, ^31^ Data regarding how many programs complied with these OPDO recommendations is limited given their recency, thus the efficacy of such interventions is yet to be formally investigated.

The otolaryngology residency application process is clearly in flux and has adapted well throughout the phases of the COVID‐19 pandemic. From this analysis of otolaryngology match trends and applicant perspectives, and an extensive review of Match process reform during the pandemic, there appears value in adopting strategies involving preference signaling and interview caps. Preference signals enable applicants to demonstrate an interest in a select few programs and encourage programs to holistically review candidates that may not have been as thoroughly considered otherwise.^25^ As only a small minority of applicants receive a large portion of interview offers, yet few complete more than 20 of those interviews,^24^ an interview cap would decrease interview hoarding to improve match rate success and optimize the alignment of program and applicant preferences.

In addition to application and selection process reform, pregraduate curriculum development and otolaryngology exposure and mentorship early on in medical school, must be considered. Opportunities such as shadowing, resident mentorship, and interest group involvement^32^ allow a greater breadth of students to explore otolaryngology as a specialty and enable departments to identify who would be “best‐fit” for the specialty. Decreasing or supplementing the emphasis on scholastic achievements in lieu of more holistic or noncognitive evaluations of applicants may attract an applicant pool better equipped to provide improved, specialty‐specific patient care.^33^

### Limitations

This study is not without its limitations. Primarily, this is an observational study in which the associations and trends presented do not necessarily imply causality. Further, due to the survey components of our study, there is a risk of recall bias, nonresponse bias, and sampling bias. As with all survey‐based research, there is potential self‐selection bias among respondents. Likewise, although our sample size is robust, our data only represent 19% of PGY‐1/PGY‐2 otolaryngology residents, thus our findings may not be generalizable to the entire otolaryngology resident population. In addition, only otolaryngology residents were contacted, thus our survey distribution method did not directly capture responses from residents who considered otolaryngology but ultimately pursued another specialty, but instead used otolaryngology residents' impressions of what factors impacted those medical school classmates' decisions. For these reasons, future studies that more comprehensively assess the factors and perceptions impacting applicants' decisions are needed.

## CONCLUSIONS

With recent changes in the USMLE Step 1 scoring system from numerical scores to pass/fail, programs may be compelled to seek new ways to differentiate applicants. However, programs must bear in mind the consequence(s) of deterring applicants when implementing any new requirement(s) for residency applications. Our study suggests that the PSP and ORTA are perceived as barriers to applying to otolaryngology and were associated with significant declines in applicant numbers and increases in match rate success. Optional PSP and postmatch ORTA conversely led to significant increases in applications and decreases in match rate success. Methods affecting application numbers should be applied strategically and with careful consideration. Though perceived barriers to otolaryngology applications may risk losing high‐quality candidates, are we really doing any better with increasing pools of unmatched applicants year after year?

## AUTHOR CONTRIBUTIONS

Neal R. Godse, Amber D. Shaffer, Noel Jabbour, Barry M. Schaitkin, and Leila J. Mady designed the work. Emma De Ravin, Neal R. Godse, Amber D. Shaffer, Noel Jabbour, Barry M. Schaitkin, and Leila J. Mady acquired and analyzed the data. Emma De Ravin, Ariel S. Frost, and Leila J. Mady drafted the original manuscript. Emma De Ravin, Ariel S. Frost, Neal R. Godse, Amber D. Shaffer, Noel Jabbour, Barry M. Schaitkin, Jason Newman, and Leila J. Mady revised and approved the final manuscript for submission. Emma De Ravin, Ariel S. Frost, Neal R. Godse, Amber D Shaffer, Noel Jabbour, Barry M Schaitkin, Jason Newman, and Leila J. Mady agree to be accountable for all aspects of the work.

## CONFLICT OF INTEREST

The authors declare no conflicts of interest.

## ETHICS STATEMENT

The above manuscript is the author's own original work, which has not been previously published and is not in submission elsewhere. The paper reflects the author's own research and analysis in a truthful and complete manner and properly credits the meaningful contributions of coauthors and coresearchers.

## Data Availability

Annual National Resident Matching Program® data are available from *Charting Outcomes in the Match* available at https://www.nrmp.org/match-data-analytics/residency-data-reports/. Postgraduate year 1/2 otolaryngology resident survey data are available from the corresponding author upon reasonable request.
